# Tannic acid-functionalized HEPA filter materials for influenza virus capture

**DOI:** 10.1038/s41598-020-78929-4

**Published:** 2021-01-13

**Authors:** Subin Kim, Jinhyo Chung, Sang Hyun Lee, Jeong Hyeon Yoon, Dae-Hyuk Kweon, Woo-Jae Chung

**Affiliations:** 1 Department of Integrative Biotechnology, Sungkyunkwan University, Suwon, Gyeonggi-do 16419 Republic of Korea; 2 Center for Biologics, Sungkyunkwan University, Suwon, 16419 Republic of Korea

**Keywords:** Health care, Materials science

## Abstract

Influenza, one of the most contagious and infectious diseases, is predominantly transmitted through aerosols, leading to the development of filter-based protective equipment. Though the currently available filters are effective at removing submicron-sized particulates, filter materials with enhanced virus-capture efficiency are still in demand. Coating or chemically modifying filters with molecules capable of binding influenza viruses has received attention as a promising approach for the production of virus-capturing filters. For this purpose, tannic acid (TA), a plant-derived polyphenol, is a promising molecule for filter functionalization because of its antiviral activities and ability to serve as a cost-efficient adhesive for various materials. This study demonstrates the facile preparation of TA-functionalized high-efficiency particulate air (HEPA) filter materials and their efficiency in influenza virus capture. Polypropylene HEPA filter fabrics were coated with TA via a dipping/washing process. The TA-functionalized HEPA filter (TA-HF) exhibits a high in-solution virus capture efficiency of up to 2,723 pfu/mm^2^ within 10 min, which is almost two orders of magnitude higher than that of non-functionalized filters. This result suggests that the TA-HF is a potent anti-influenza filter that can be used in protective equipment to prevent the spread of pathogenic viruses.

## Introduction

The influenza A virus (IAV) is one of the most contagious and rapid spreading infectious diseases. Every year, 1 billion cases are reported, of which 3‒5 million are severe cases, with 290,000‒650,000 influenza-related respiratory deaths worldwide^1^. Aerosol transmission is the predominant mode of influenza virus transmission in households, accounting for approximately half of all transmission events^2^. Thus, preventive measures to deal with influenza virus particles present in the air are urgently needed. To eliminate airborne viruses, filters have been developed for face masks, air purifiers, and ventilation systems. High-efficiency particulate air (HEPA) filters in particular are gaining popularity as they block the passage of approximately 99.97% of particles with a diameter > 0.3 μm^3^. However, viral particles with a small diameter (e.g., influenza virus; ~ 100 nm) may still pass through the filter^3^. Therefore, filters with enhanced virus capture capability based on affinity binding, which would efficiently limit the spread of viral infection, are in high demand. Several types of antiviral and virus-adsorbing materials have been incorporated in or coated on filters, including sialyllactose^4^, curdlan sulfate^5^, poly(ethylenimine)^6^, and sodium chloride^7^. Although previous reports have shown promising results for viral capture or inactivation, they have limitations such as high cost^4,5^, narrow-spectrum targets^4^, multi-step production^4,5^, possible toxicity^6^, or insufficient stability^7^. Particularly during pandemics, it is crucial to supply cheap and easily made respirators (e.g., filters and masks) rather than expensive and complex ones, so that even countries with economical constraint do not suffer from severe shortages of equipment. Thus, it is important to develop cost-effective and environmentally friendly coating materials that can serve as broad-spectrum virus capturing agents on filter surfaces with high efficiency and stability. Tannic acid (TA), a high molecular weight polyphenol, is widely known for its antioxidant and antibacterial activities^8^ and protein-aggregating properties^9,10^. TA has a unique structure with multiple catechol and galloyl groups linked to a glucose core. This structure allows it to form multiple modes of interaction, such as hydrogen bonding, metal chelation, and electrostatic and hydrophobic interactions, with a variety of materials of different chemical compositions and surface properties^11–16^. In addition, owing to the recent outbreak of novel respiratory disease viruses, the antiviral activity of polyphenols against a wide range of virus strains such as herpes virus^17^, Zika virus^18^, coronavirus^19,20^, and influenza virus^21,22^ have been highlighted. In particular, TA has been spray-coated on filter materials to inactivate influenza viruses, as described in a previously published patent^23^. However, several important issues involved in the development of TA-treated filter materials have not been thoroughly addressed, such as the virus capture property, storage stability, and mechanistic basis of virus infection inhibition.

**Figure 1:**
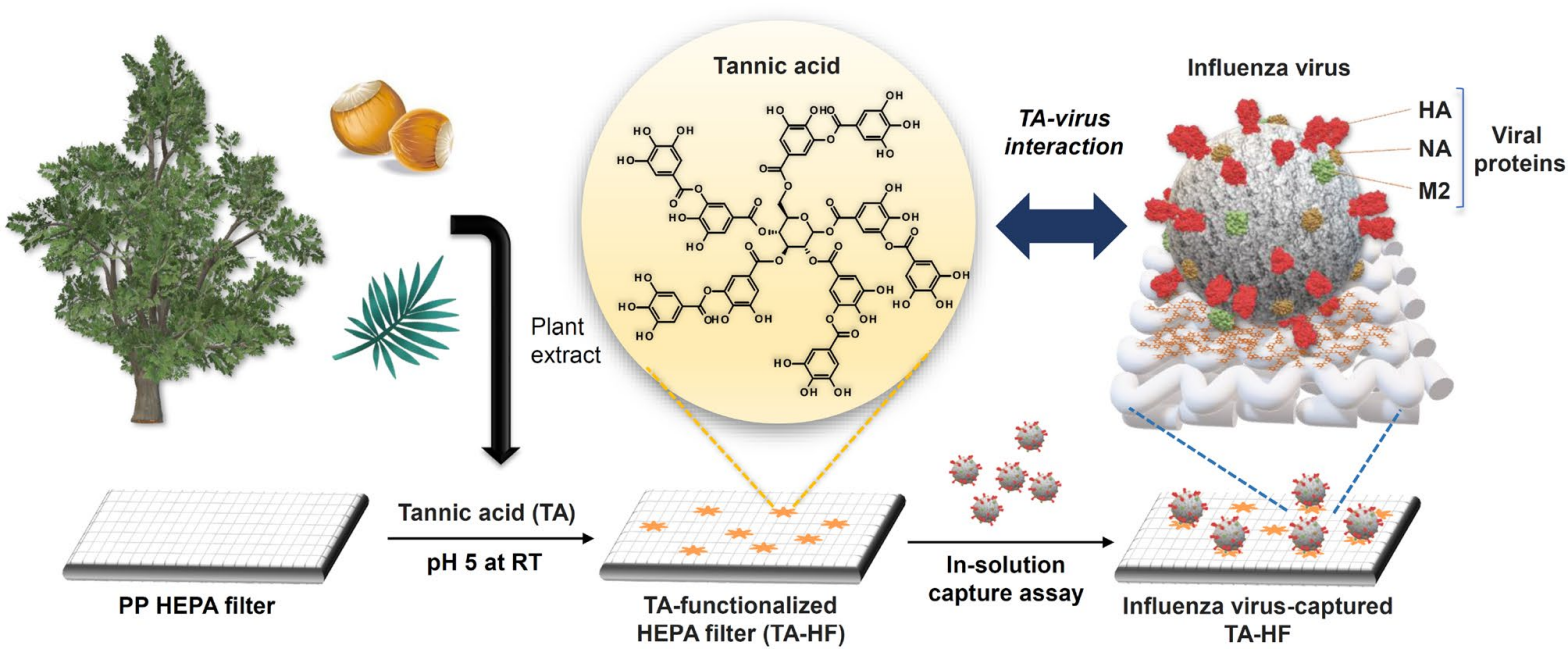
Preparation of TA-HF for influenza virus capture. Schematic illustration of TA-HF preparation and influenza virus capture based on interaction of TA with viral proteins (*HA* hemagglutinin, *NA* neuraminidase, *M2* matrix-2). *TA* tannic acid, *HF* high-efficiency particulate air filter.

In this study, we demonstrate a simple, cost-effective, and robust strategy for anti-influenza filter development by introducing the natural antiviral compound TA onto the surface of polypropylene (PP) HEPA filter fabric, combining the material-independent coating and virus-capturing properties of TA (Fig. 1). We also demonstrate a possible mechanism for TA-functionalized filter materials to capture influenza viruses and their potential for practical use based on results from inhibition assays, cytotoxicity assays, assessing storage stabilities at different temperatures, and monitoring capturing capability in typical environments where these filters would be used.

## Results and discussion

### Preparation and characterization of TA-functionalized HEPA filter materials

**Figure 2:**
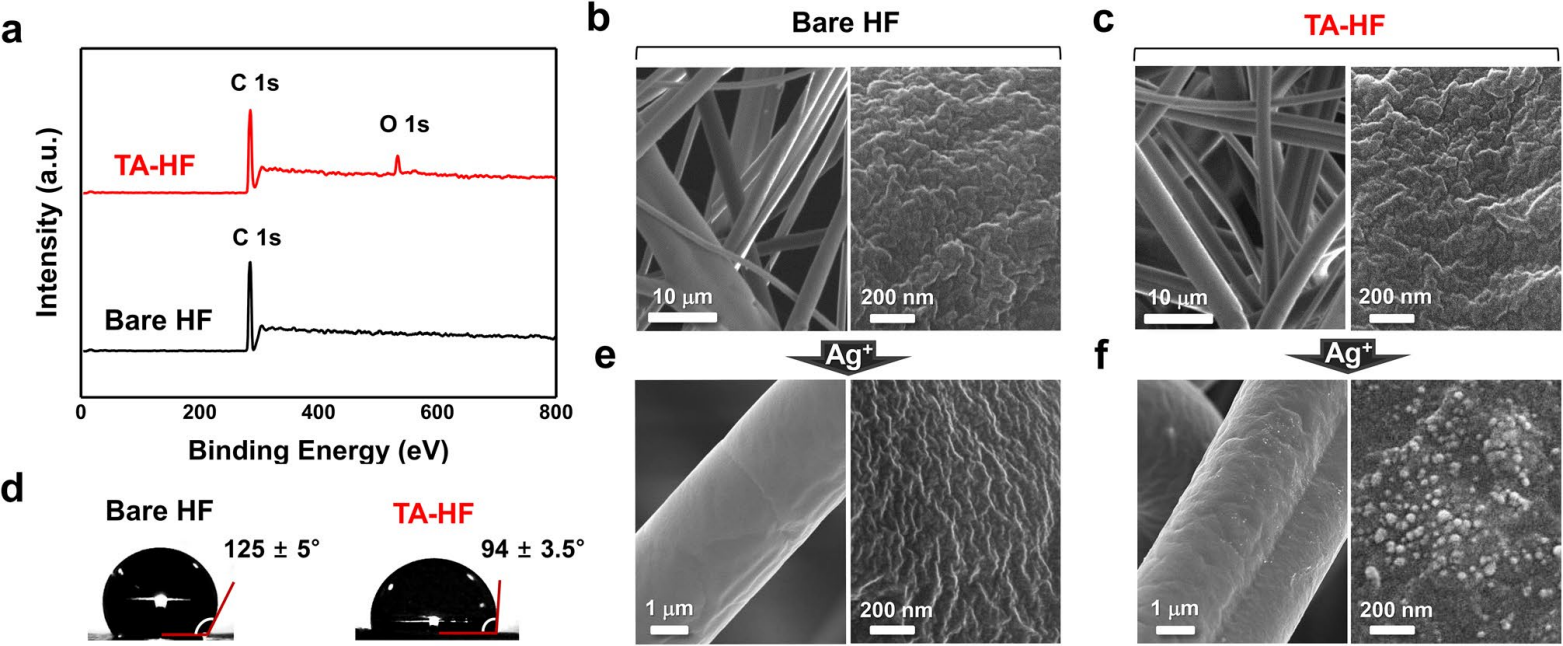
Surface characterization of TA-HF. **(a)** XPS spectra of TA-HF (top) and bare HF (bottom). **(b,c)** SEM images showing the surface morphologies of the bare HF **(b)** and TA-HF **(c)**. The images on the left are at lower magnification, and the images on the right are at higher magnification. **(d)** Water contact angle images of the bare HF (left) and TA-HF (right). **(e,f)** SEM images of the bare HF **(e)** and TA-HF **(f)** after silver nitrate treatment (100 mM, 12 h, 22 °C). *TA* tannic acid, *HF* high-efficiency particulate air filter, *SEM* scanning electron microscopy.

To prepare TA-functionalized HEPA filter fabric (TA-HF), the filter was treated with TA solution (1, 5, 10, and 20 mg/mL, pH 5) (Fig. 1). TA functionalization was confirmed by X-ray photoelectron spectroscopy (XPS). The only peak observed for the bare HF XPS spectrum was C 1s, as the PP hydrocarbon surface contained no heteroatoms (Fig. 2a, bottom). However, an O 1s peak appeared after TA functionalization (Fig. 2a, top), indicating that TA was successfully introduced to the PP surface. We confirmed that the peak area ratio O 1s/C 1s of the filter materials increased from 0.09 to 0.12 as the TA concentration increased from 1 to 5 mg/mL (Fig. S1). Moreover, in the TA concentration range of 5–20 mg/mL, O 1s/C 1s remained relatively constant at 0.12–0.13 (Fig. S1). Therefore, a TA concentration of 5 mg/mL was employed in further experiments. Even after TA functionalization, no significant change was observed in the porous fibrous structures and surface morphology of the HF (Fig. 2b,c). Meanwhile, the HF surface became less hydrophobic after TA functionalization, which was confirmed by water contact angle measurements (Fig. 2d). The TA-HF surface showed a decreased contact angle of 94 ± 3.5° compared to a contact angle of 125 ± 5° for the bare HF surface, again confirming that hydrophilic TA is present on the HF surface. The phenolic functionality of TA on TA-HF was examined by assessing nanoparticle growth in the presence of silver ions, taking advantage of the reducing properties of TA. The growth of nanoparticles was observed on TA-HF after immersion in AgNO_3_ solution (100 mM) for 12 h, but no nanoparticles were observed on the bare HF (Fig. 2e,f). These nanoparticles were identified as silver-based on energy-dispersive X-ray spectroscopy (EDS) (Fig. S2).

### TA-hemagglutinin interaction

**Figure 3:**
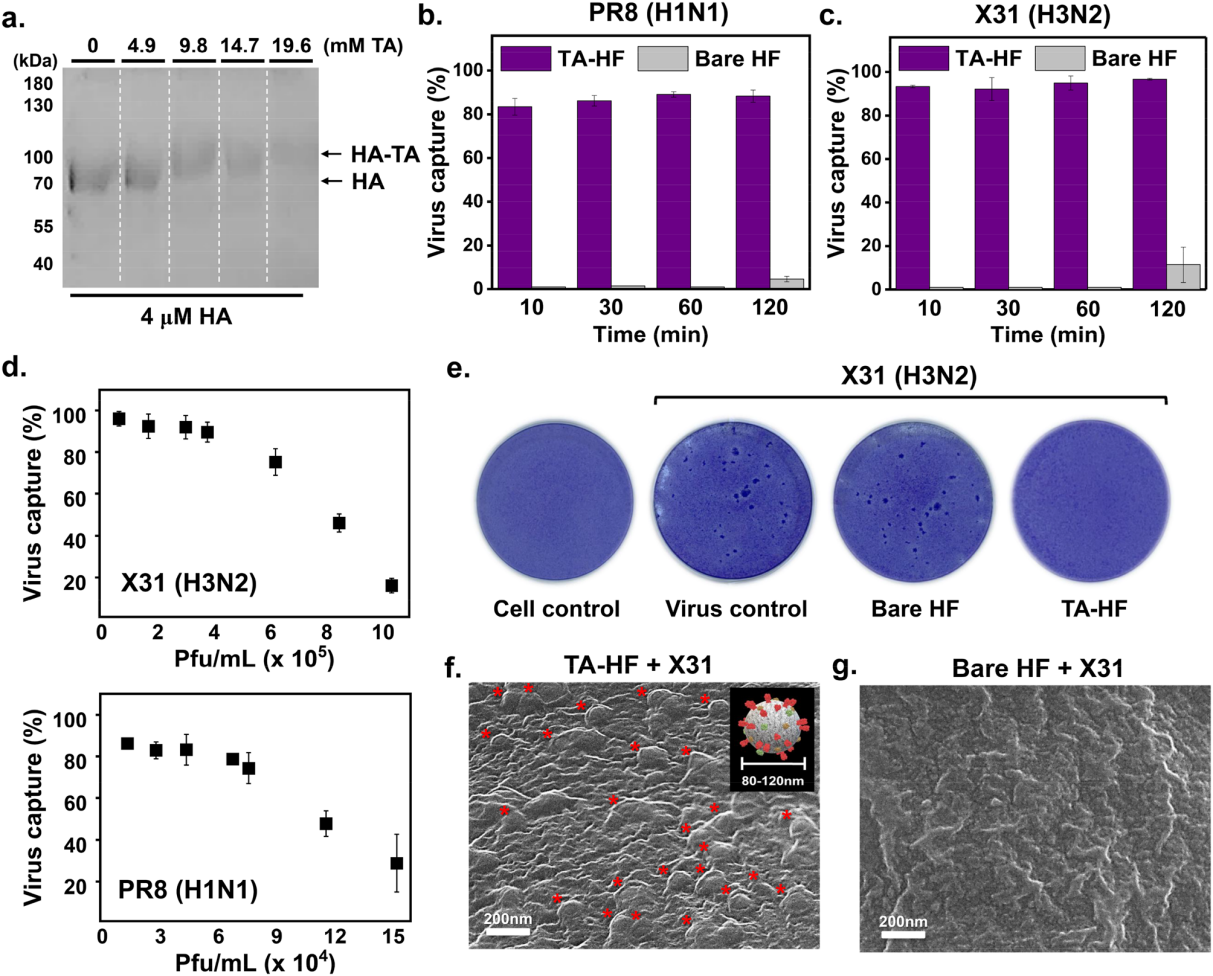
Influenza virus capture efficiency of TA-HF. **(a)** SDS-PAGE of hemagglutinin (HA: H1N1) pre-treated with different concentrations of TA (0–19.6 mM). HA (4 μM) was treated with TA for 2 h at 22 °C. **(b,c)** Influenza A virus (IAV) capture (%) by TA-HF and bare HF after 10, 30, 60, and 120 min incubation with PR8 (H1N1) **(b)** and X31 (H3N2). Viral titer was measured by the MUNANA assay. **(d)** IAV capture (%) by TA-HF and bare HF after incubation with different concentrations of X31 (top) and PR8 (bottom). **(e)** Evaluation of the virus capture efficiency of the bare HF and TA-HF by plaque reduction assay. MDCK cells were incubated with X31, which had been incubated with TA-HF, bare HF, or left untreated (virus control). **(f,g)** SEM images of the TA-HF **(f)** and bare HF **(g)** after incubation with X31. *TA* tannic acid, *HF* high-efficiency particulate air filter, *SEM* scanning electron microscopy, *IAV* influenza A virus.

We examined whether TA molecules exhibit binding to hemagglutinin (HA), the most abundant protein on the surface of influenza viruses. The HA-TA interaction was observed by SDS-PAGE, where the HA band at approximately 70 kDa shifted to a higher molecular weight, and its intensity became weaker as the concentration of TA increased from 4.9 to 19.6 mM (Fig. 3a and Fig. S2). The shift and intensity decrease of the band were accompanied by TA-HA aggregate formation, which remained in the well of the gel (Fig. S3). This result indicates that the TA-HF material may mediate adsorption of IAV onto the filter surface via TA-HA interaction.

### Efficiency of the TA-HF in influenza virus capture

Indeed, we observed that TA-HF efficiently captured IAVs such as A/Puerto Rico/8/1934 H1N1 (PR8) and A/Aichi/2/68 H3N2 (X31) from suspension within 10–120 min, while the bare HF removed only 11% and 5% of X31 and PR8, respectively, even after 120 min (Fig. 3b,c). Each filter sample was prepared as a circular disk with a diameter of 6 mm and placed in a viral suspension [230 μL, 12,236‒83,720 plaque-forming units (pfu)] for 10 or 120 min with gentle shaking, followed by a neuraminidase activity assay of the remaining suspension to determine the viral concentration based on the standard curve (Fig. S4). TA-HF captured most of the viral particles within 10 min [PR8, 83% (345 pfu/mm^2^); X31, 93% (2723 pfu/mm^2^)], indicating that IAV capture by TA-HF occurs rapidly. Following this, we investigated the IAV concentration-dependent capture efficiency of TA-HF. Capture efficiencies of > 80% and > 90% were observed for PR8 and X31, respectively, in the high viral concentration range of 4 × 10^4^ pfu/mL to 4 × 10^5^ pfu/mL (Fig. 3d). These results revealed that the full capacity of TA-HF to capture virus was 14,187 pfu/mm^2^ for X31 and 1,968 pfu/mm^2^ for PR8 (Fig. S5). The remarkable IAV capturing capability of TA-HF was also confirmed by the reduction in plaque formation of the viral suspensions that had been in contact with TA-HF (Fig. 3e and Fig. S6). The bare HF reduced the plaque number by only 8.9‒28.5%, whereas TA-HF successfully eliminated all plaques (similar to the cell-only control). IAV capture was clearly observed using scanning electron microscopy (SEM) (Fig. 3f and Fig. S7 (right)); spherical virus particles (100‒120 nm in diameter) were observed on the TA-HF filter, whereas the bare HF did not show viral adsorption (Fig. 3g and Fig. S7 (left)). We believe that the virus-capturing efficiency of TA-HF shown in these in-solution tests can be translated to that of TA-HF for aerosols or droplets containing respiratory fluid and viral particles in the following aspect. Viral aerosols are tiny particles of liquid that contain viruses floating in the air. Therefore, upon contact of viral aerosols with filters, interaction between the virus and filter surface occurs at the interface between the filter surface and the moist liquid.

### Cytotoxicity and storage stability of TA-HF

**Figure 4:**
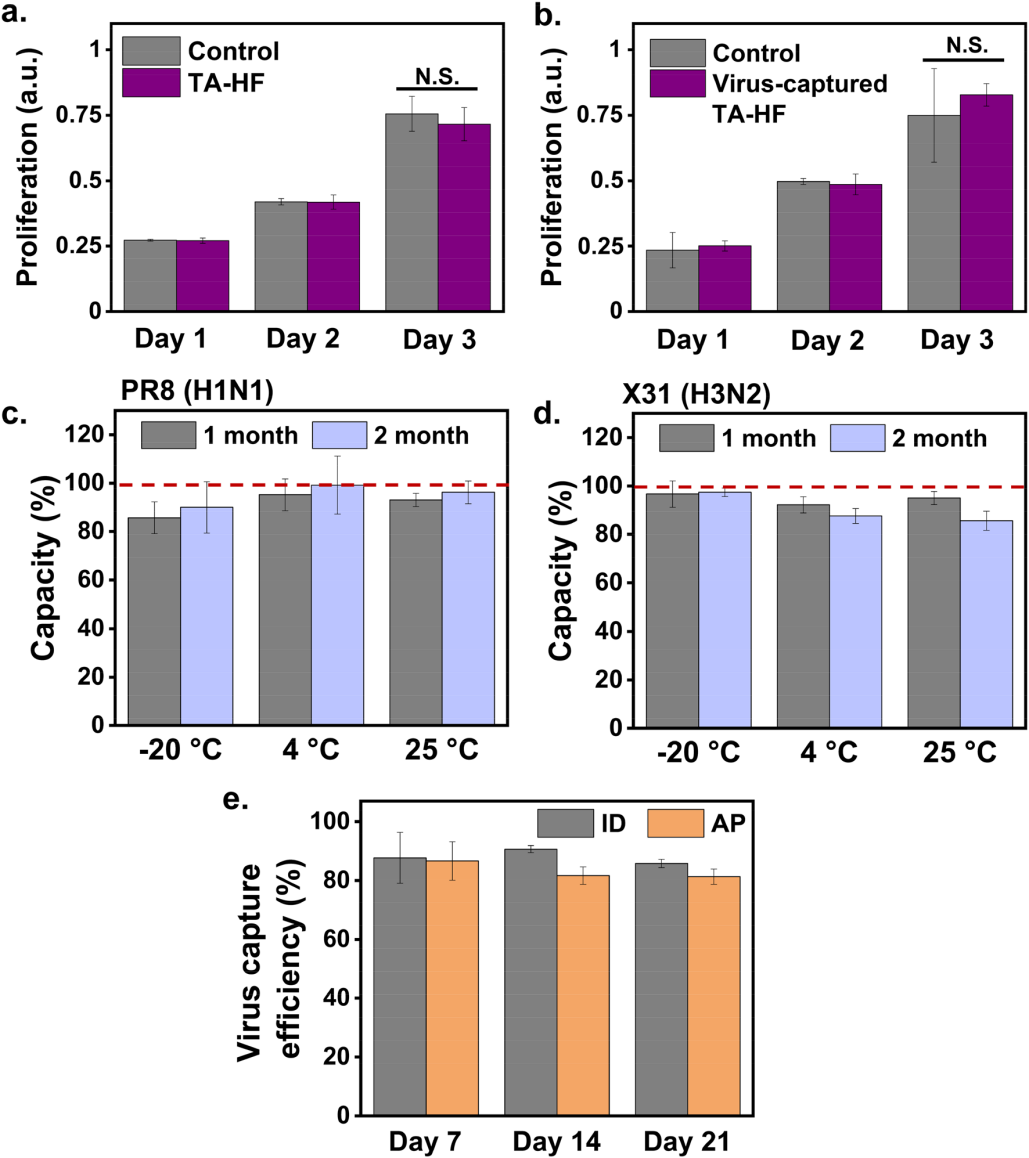
Investigation of the biosafety and storage stability of TA-HF. **(a,b)** Cell viability assay to assess the cytotoxicity of TA-HF (n = 3) before **(a)** and after influenza virus capture **(b)**. **(c,d)** Influenza virus capture efficiency of TA-HFs, which were freshly prepared and stored for one or two months at − 20 °C, 4 °C, or 25 °C, to determine the storage stability of TA-HF. Red dotted lines indicate initial virus-capture capacity of TA-HF. **(e)** Influenza virus capture efficiency of TA-HFs for a period of three weeks placed in indoor environment (ID) and inside an air purifier environment (AP). *TA* tannic acid, *HF* high-efficiency particulate air filter.

For practical use of the developed filter materials, it is important to investigate their biosafety and storage stability properties. A cell viability assay performed using a Transwell system showed that TA-HF exhibited no cytotoxicity toward Madin-Darby canine kidney (MDCK) cells over a period of 3 days (Fig. 4a). Next, a TA-HF sample used for X31-capture was immediately tested for cytotoxicity in the same manner, to examine whether the captured viruses can exert a negative effect on cell growth (Fig. 4b and Fig. S8). The captured viruses exhibited no detectable cytotoxicity, indicating that they remained firmly attached to the filter surface and were not released into the culture medium. The storage stability of TA-HF was evaluated by measuring the virus capture efficiency of filters after storage at − 20 °C, 4 °C, or 25 °C for one or two months. Temperature had no impact on the stability of the filters over the storage period (Fig. 4c,d), and the virus capture efficiencies of the TA-HFs remained in the range of 87‒99% after two months of storage.

### Evaluation of filter lifetime in a typical environment

We designed experiments to monitor changes in the lifetime of the filters placed in a virtual regular environment where the filters would possibly be used. Two locations, indoor (ID) group and an air purifier (AP) group, were chosen. The ID group was a typical indoor office environment where several people spent a majority of their time per day. In the AP group, a filter was placed inside an air purifier between a pre-filter and HEPA filter, both of which were in the purifier. The air purifier was placed in the aforementioned indoor office and set to operate for a period of 21 days. The TA-HF filter in each group was removed at designated time points (0, 7, 14, and 21 days) and immediately assayed for in-solution virus capture. The TA-HFs in both ID and AP groups retained their virus-capturing efficiency up to 85% (ID) and 81% (AP) for 21 days (Fig. 4e). These results indicate that the TA-functionalized filters have immense potential for practical use in influenza virus prevention equipment and devices.

## Conclusions

The simple and environmentally friendly process of TA-HF preparation was demonstrated, and the TA-HF showed remarkable efficiency for IAV capture based on the strong interaction between TA and IAV. Rapid and efficient virus capture was achieved by TA-HF, best exemplified by the capture performance of 2730 pfu/mm^2^ for the X31 virus in a span of 10 min. TA-HF displayed no cytotoxicity and was stable enough to retain over 87% of the initial virus capture capability during a two-month storage, which holds great promise for practical use in influenza virus infection-preventive filters. The use of versatile TA as a filter coating material allows not just the development of highly affordable and easy-to-produce anti-influenza filters, but also demonstrates that TA-functionalized filters may be expanded to prevent the spread of broad-spectrum pathogenic viruses.

## Methods

### Materials

PP HEPA filter fabrics (H14) were gifts from Envioneer Co., Ltd (Sungnam, Korea). All reagents were of analytical grade and used as received without further purification. Acetic acid, sodium acetate, silver nitrate (AgNO_3_), phosphate buffered saline (PBS), tosyl phenylalanyl chloromethyl ketone (TCK)-treated trypsin, minimum essential medium (MEM), and antibiotic/antimycotic solution (Pen/Strep/Fungiezone) were purchased from Hyclone (Pittsburgh, USA). TA, 36.5% formaldehyde, and crystal violet were purchased from Sigma Aldrich (USA). Polypropylene (PP) nominal prefilter (0.2 μm) was purchased from Sterlitech (Korea). Madin-Darby canine kidney epithelial cells (MDCK, NBL-2) were obtained from the American Type Culture Collection (ATCC) (USA). Dulbecco’s modified Eagle’s medium (DMEM) was purchased from Thermo Fisher (Waltham, MA, USA), and 4-methylumbelliferyl-N-acetyl-α-D-neuraminic acid (MUNANA) was purchased from GoldBio (St Louis, MO, USA). Sea plaque agarose was purchased from Lonza (Alpharetta, GA, USA). Cell counting kit-8 (CCK-8) was purchased from Dojindo Molecular Technologies, Inc. (Japan). Fetal bovine serum (FBS) was obtained from Atlas Biologicals (Fort Collins, CO, USA).

### Preparation of TA-HF

TA (0.1 g) was dissolved in 20 mL of pH 5.0 acetate buffer. The fabric of the HEPA filter was cut into a rectangle of 2 cm × 4 cm and immersed in the TA solution. The TA coating proceeded for 16 h at 22 °C in the dark with gentle shaking. After the functionalization process, the filter fabric was transferred to fresh deionized water and washed three times (15 mL each). The excess water was removed using filter paper and gaseous nitrogen and dried in air for 12 h. For XPS, a PP nominal prefilter (0.2 μm) was used because the fabric of the HEPA filter was not appropriate for XPS due to its loosely woven texture. All coating, washing, and drying steps of the PP nominal prefilter were performed in the same manner as described above.

### Characterization of TA-HF

The surface morphology and chemical composition of the prepared TA-HF were analyzed by field emission scanning electron microscopy (FE-SEM) and XPS. The TA coating was also confirmed by silver nanoparticle growth on the filter, which took advantage of the reducing property of TA. The filter samples were analyzed by FE-SEM (JSM7000F, JEOL, Japan) after coating with iridium to a thickness of 3 nm. The accelerating voltage was 5 kV. The typical magnification was × 50,000‒100,000. Elemental mapping was conducted with SEM (JSM7000F, JEOL, Japan) to observe silver nanoparticle formation on TA-HF samples. XPS analysis of the filter fabrics was performed using a Model ESCALAB250 (Thermo Scientific, USA) instrument with a monochromatic Al Kα X-ray source. For each sample, a survey scan from 0 to 1100 eV binding energy (BE) and elemental scans of O 1s and C 1s were acquired to determine the composition. Measurements were performed on three independent replicates for each sample. The growth of silver nanoparticles on the filter samples was initiated by immersing the TA-HF in aqueous AgNO_3_ (100 mM) and incubating it in the dark at 22 °C for 12 h. The resulting filters were washed with deionized water three times and dried using gaseous nitrogen prior to SEM analysis. The contact angle was measured by angle analysis in ImageJ (http://rsb.info.nih.gov/ij/) using an image captured with a digital microscope (AM7013MZT, Dino-Lite, USA). For each drop, clean deionized water was used, and the drop volume was 10 μL. The final measurement was an average of 3 replicates for each drop.

### Viral harvest and purification

Fertile eggs were incubated in a brooder at 37 °C and 45% humidity for 10 days. Then, the A/Puerto Rico/8/1934 H1N1 (PR8) or A/Aichi/2/68 H3N2 (X31) strain of the influenza virus was inoculated into the eggs at 10,000 pfu/mL (100 μL for each egg). The development of the eggs was aborted 3 days post-infection by moving to a refrigerator at 4 °C. The next day, the allantoic fluid was collected from each egg and centrifuged at 1792 × *g* for 30 min to remove excess debris. The supernatant was filtered through a 0.45 μm pore size filter and centrifuged at 36,042 *g* for 1.5 h. Each pellet was resuspended in 1 mL of PBS (pH 7.4) overnight. The following day, the viral suspensions were purified by sucrose gradient centrifugation (50%, 40%, 30%, and 20%, sequentially from bottom to top) at 58,804 × *g* for 2.5 h. The band that appeared between the 40% and 30% layers was collected. After additional centrifugation at 58,804×*g* for 1.5 h, the virus pellet was resuspended in PBS at an appropriate titer. The final virus stock was snap-frozen in liquid nitrogen and stored at − 80 °C in a deep freezer.

### IAV tittering based on neuraminidase activity assay

The viral suspension (200 μL) was placed in a well of a black 96-well plate. Then, 50 μL of MUNANA (0.5 mM in PBS) was added to each well. Following incubation of the mixture at 37 °C for 2 h, the resulting fluorescence of the mixture was measured (λ_ex_: 355 nm; λ_em_: 460 nm). The fluorescence values were converted to PFU based on the standard curve for each viral strain (Fig. S3).

### Plaque formation reduction assay

MDCK cells were seeded at a density of 3 × 10^5^ cells/well in a 12-well plate. When cells reached 90‒100% confluency, the cells were washed with PBS and inoculated with PR8 or X31 for 1 h. The cells were then rinsed with PBS and overlaid with low-melting-temperature agar. After 3‒4 days, the cells were fixed with 1 mL of 0.38% formaldehyde for 3 h. Then, the agar overlay was carefully removed with running water and stained with 0.5% crystal violet for 30 min. For in-solution virus-capture assay, viral suspension (PR8: 2.56 × 10^6^ pfu/mL; X31: 3.36 × 10^6^ pfu/mL) that had been in contact with TA-HF or bare HF for 1 h was diluted 5 × 10^4^ times, and 0.5 mL of the resulting viral suspension was used as inoculum. The number of plaque forming units (pfu)/mL was calculated according to the following equation: number of plaques/[dilution factor × (mL of inoculum/plate)].

### In-solution virus-capture assay

PR8 and X31 suspensions were diluted with PBS to a designated concentration. Then, the in-solution virus capture assay was performed in a 96-well round plate (230 μL in each well). Each filter sample was prepared as a circular disk with a diameter of 6 mm using a biopsy punch and placed in the viral suspension for 10 or 120 min with gentle shaking. Then, the filter samples were washed three times with PBS, and the titer of the remaining influenza virus suspension was evaluated based on MUNANA or plaque assay.

## Supplementary Information

Supplementary Information.
