# Supplementary material for: Tannic acid-functionalized HEPA filter materials for influenza virus capture
Source: Sci Rep. 2021 Jan 13;11:979. doi: 10.1038/s41598-020-78929-4 (PMC7806633; doi:10.1038/s41598-020-78929-4)
Supplement: Supplementary file 1 — Supplementary Information. [file 41598_2020_78929_MOESM1_ESM.pdf]

Supplementary Information

Tannic acid-functionalized HEPA filter materials for influenza virus capture

Subin Kim, Jinhyo Chung, Sang Hyun Lee, Jeong Hyeon Yoon, Dae-Hyuk Kweon, Woo-Jae Chung*

Supplementary methods

SDS-PAGE-based analysis of HA-TA interaction

Loading samples were prepared by mixing 4 μM HA and 4.9 mM, 9.8 mM, 14.7 mM, or 19.6 mM TA in equal volume, and were incubated for 2 h with gentle shaking. Then 6x sample buffer was added, following which the mixture was heated at 100 °C for 5 minutes, and resolved on a 12% SDS-PAGE; protein markers were run simultaneously with the samples. The gel was stained with 0.1% Coomassie blue R250 for 4 h, and then destained overnight until each band became distinct from the background.

Cytotoxicity assay for TA-HF and virus-captured TA-HF

The cytotoxicity of TA-HF was measured using CCK-8 according to the manufacturer's instructions. MDCK cells were seeded at a density of 2×10^4 cells/well in a 12-well plate and incubated overnight in MEM containing 10% FBS and antibiotics. After incubation for 3 days, the absorbance of each well was measured at 450 nm using a microplate reader. For virus-captured TA-HF, the filter was placed in 10,310 PFU/230 μL PR8 virus solution for 2 h,

washed three times with deionized water, and transferred to a 12 Transwell cell culture plate (Diam. 6.5 mm, pore size 8.0 μm).

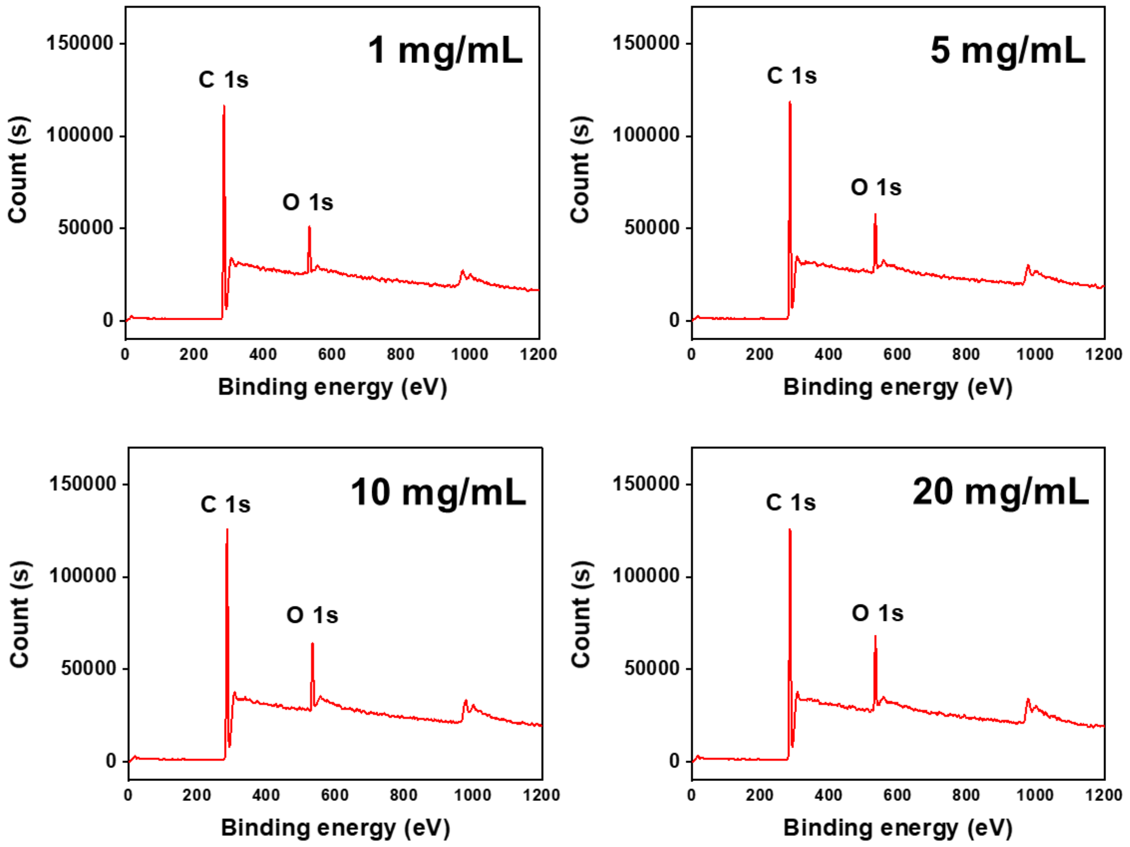

TA concentration	Atomic content (%)		
	C 1s	O 1s	O 1s/C 1s
1 mg/mL	91.72	8.28	0.090
5 mg/mL	89.46	10.54	0.118
10 mg/mL	89.14	10.86	0.122
20 mg/mL	88.42	11.58	0.131

Figure S1. XPS spectra and composition analysis of TA-HF prepared using different TA concentration.

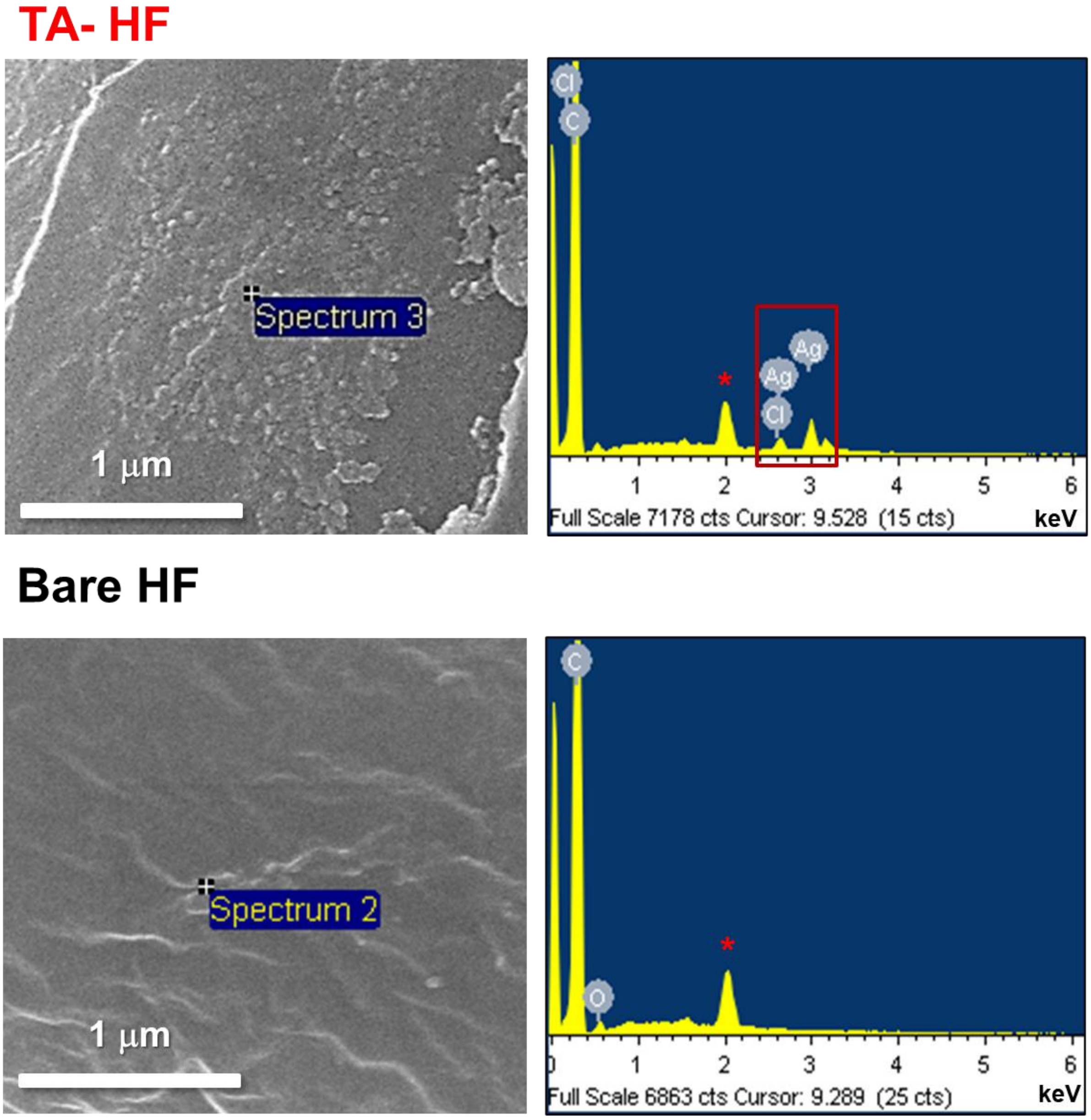

Figure S2. Energy dispersive X-ray spectroscopy of Ag nanoparticles grown on TA-HF (top) and bare HF (bottom). Ag peak at 2.984 keV was appeared on TA-HF showing that spherical forms are Ag nanoparticles. The peaks assigned as * are iridium, the coating materials for sample preparation.

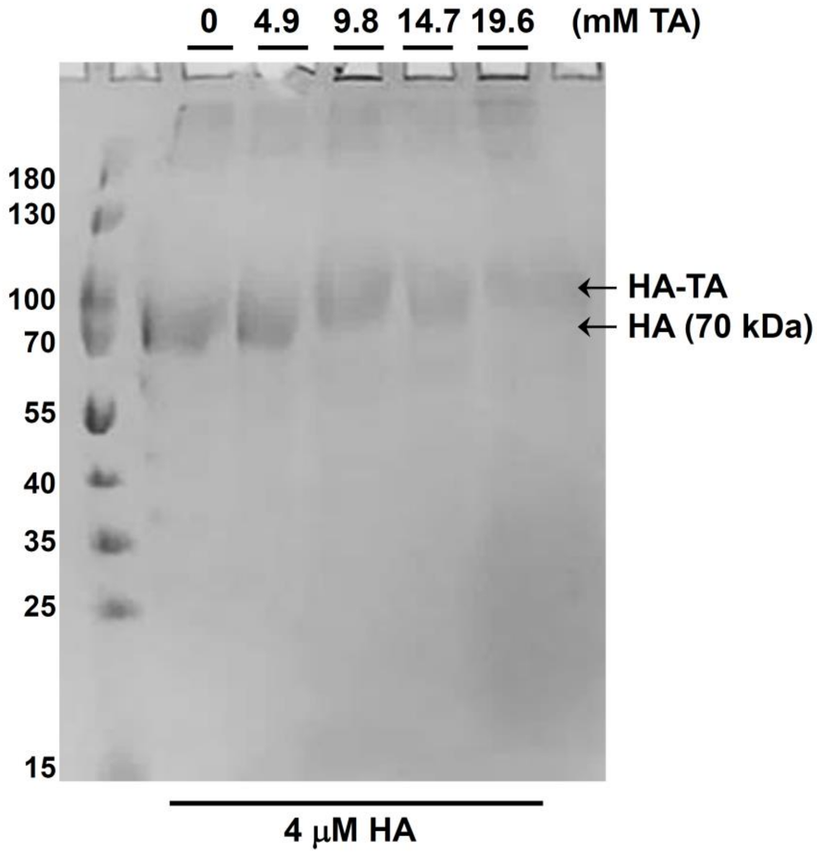

Figure S3. SDS-PAGE of hemagglutinin (HA: H1N1) pre-treated with different concentrations of TA (0-19.6 mM). HA (4 μM) was treated with TA for 2 h at 22 °C. Each sample was resolved on a 12% SDS-PAGE after heating at 100 °C for 5 minutes.

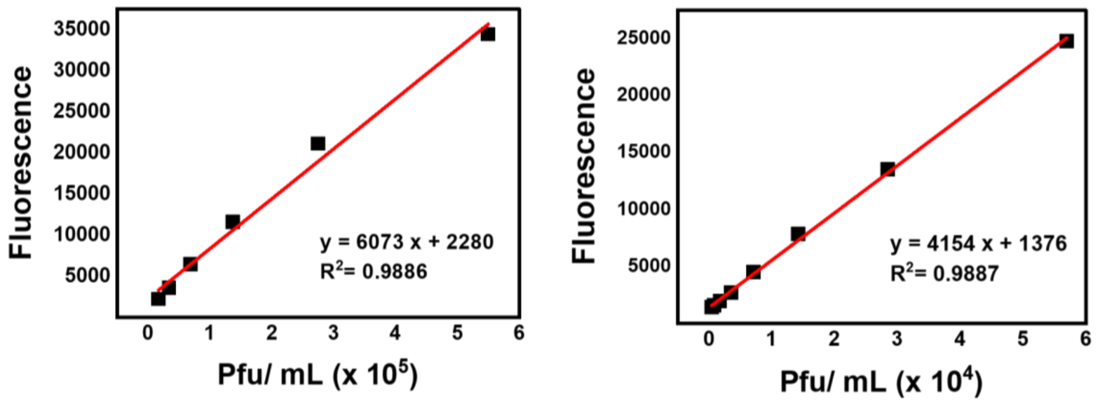

Figure S4. Standard curves for fluorescence MUNANA-based assay: X31 (left) and PR8 (right).

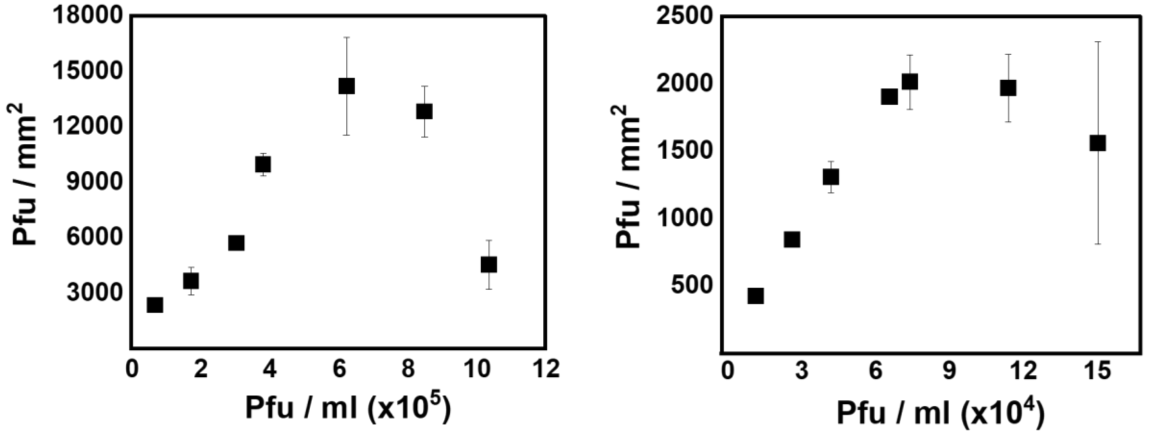

Figure S5. Virus-capture capacity of TA-HF to X31(left) and PR8 (right).

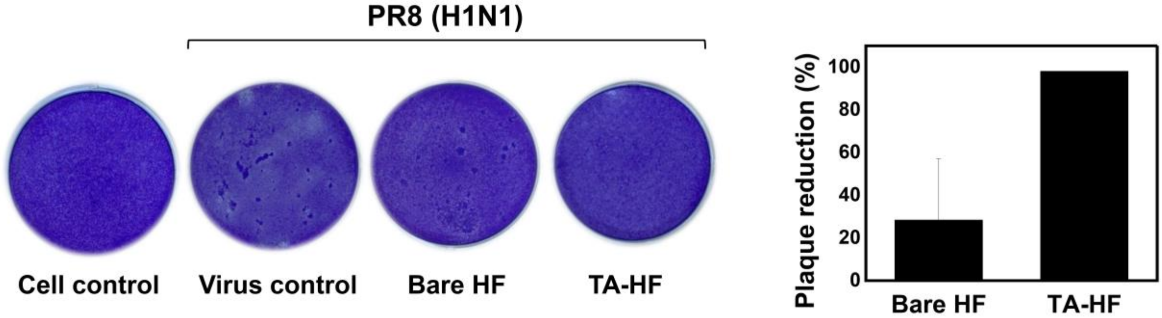

Figure S6. Evaluation of virus capture efficiency of the bare HF and TA-HF by plaque reduction assay. MDCK cells were incubated with PR8 which had been either pre-treated with each filter or left untreated.

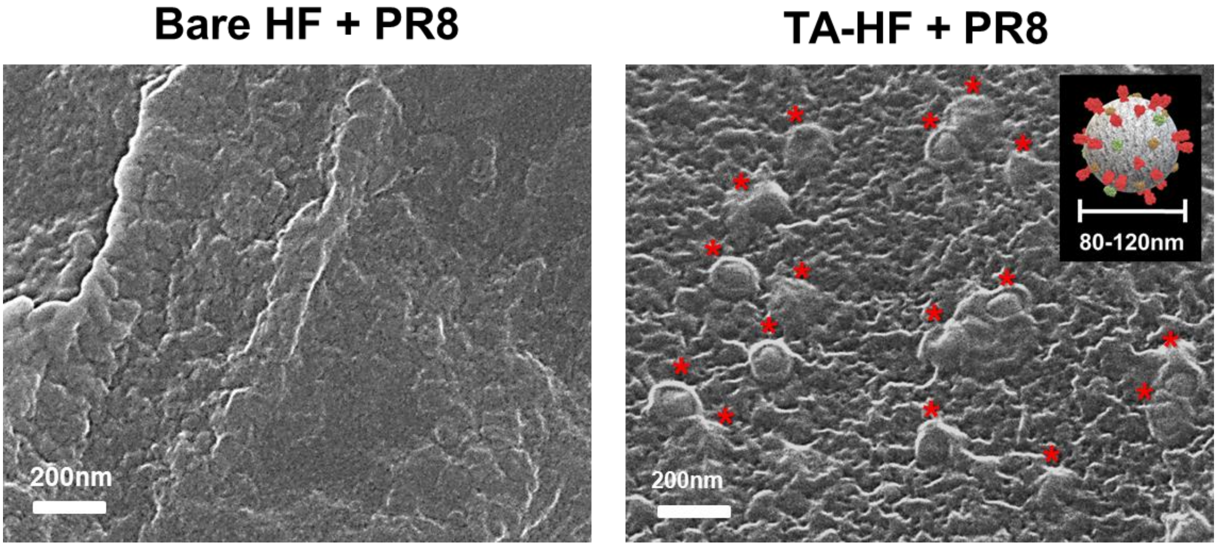

Figure S7. FE-SEM image of bare HF and TA-HF incubated in viral suspension. Concentration of virus suspension was 1.96×10^5 pfu/230 μL. Red asterisks indicate the viral particles.

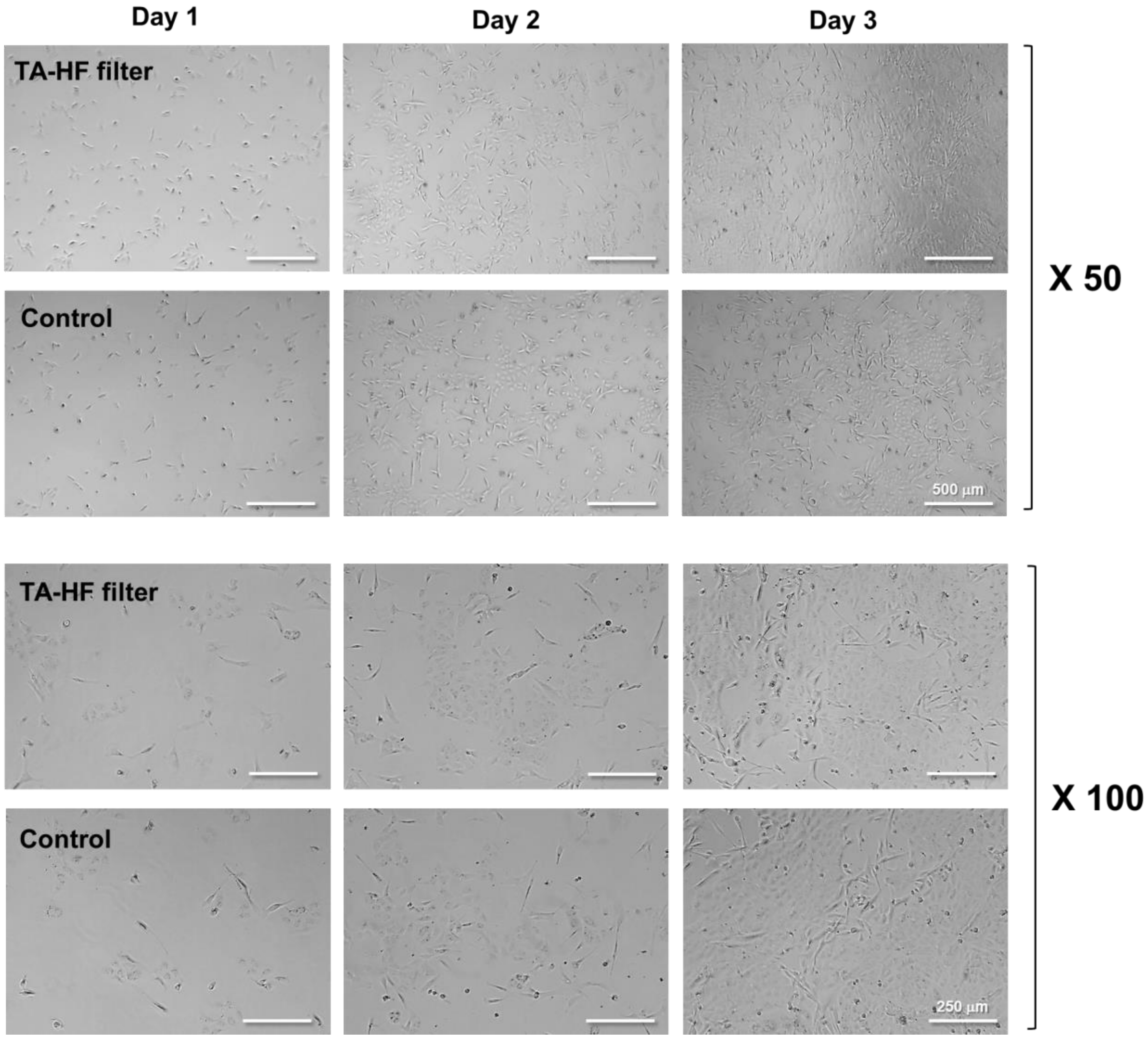

Figure S8. Growth of MDCK cells with and without X31-captured TA-HF. MDCK cells were incubated for 3 days. Both control and TA-HF showed similar cell growth.
